# SmartSkin-XAI: An Interpretable Deep Learning Approach for Enhanced Skin Cancer Diagnosis in Smart Healthcare

**DOI:** 10.3390/diagnostics15010064

**Published:** 2024-12-30

**Authors:** Sultanul Arifeen Hamim, Mubasshar U. I. Tamim, M. F. Mridha, Mejdl Safran, Dunren Che

**Affiliations:** 1 Department of Computer Science, American International University-Bangladesh, Dhaka 1229, Bangladesh; arifeenhamim@gmail.com (S.A.H.); mubassharul310@gmail.com (M.U.I.T.); 2 Department of Computer Science, College of Computer and Information Sciences, King Saud University, P.O. Box 51178, Riyadh 11543, Saudi Arabia; 3 Department of Electrical Engineering and Computer Science, Texas A&M University-Kingsville, Kingsville, TX 78363, USA; dunren.che@tamuk.edu

**Keywords:** melanoma detection, explainable AI, deep learning, DenseNet, SmartSkin-XAI

## Abstract

**Background:** Skin cancer, particularly melanoma, poses significant challenges due to the heterogeneity of skin images and the demand for accurate and interpretable diagnostic systems. Early detection and effective management are crucial for improving patient outcomes. Traditional AI models often struggle with balancing accuracy and interpretability, which are critical for clinical adoption. **Methods:** The SmartSkin-XAI methodology incorporates a fine-tuned DenseNet121 model combined with XAI techniques to interpret predictions. This approach improves early detection and patient management by offering a transparent decision-making process. The model was evaluated using two datasets: the ISIC dataset and the Kaggle dataset. Performance metrics such as classification accuracy, precision, recall, and F1 score were compared against benchmark models, including DenseNet121, InceptionV3, and esNet50. **Results:** SmartSkin-XAI achieved a classification accuracy of 97% on the ISIC dataset and 98% on the Kaggle dataset. The model demonstrated high stability in precision, recall, and F1 score measures, outperforming the benchmark models. These results underscore the robustness and applicability of SmartSkin-XAI for real-world healthcare scenarios. **Conclusions:** SmartSkin-XAI addresses critical challenges in melanoma diagnosis by integrating state-of-the-art architecture with XAI methods, providing both accuracy and interpretability. This approach enhances clinical decision-making, fosters trust among healthcare professionals, and represents a significant advancement in incorporating AI-driven diagnostics into medicine, particularly for bedside applications.

## 1. Introduction

Skin cancer is one of the fastest-growing health problems worldwide, caused by the uncontrolled growth of abnormal skin cells. It is one of the most common and serious cancers globally, and therefore it is considered a significant public health issue [1]. Melanoma is the most dangerous form of skin cancer, with a high potential to metastasize to other organs if treatment is not initiated early. Although non-melanoma skin cancers, such as basal cell carcinoma, are usually not aggressive, melanoma is much more dangerous because it tends to metastasize quickly [2]. The most important risk factor for skin cancer is exposure to ultraviolet (UV) radiation from the sun, which causes DNA damage in skin cells. Other risk factors include light-colored skin, a history of sunburn, excessive sun exposure, living in sunny or high-altitude regions, precancerous skin lesions, and a family history of skin cancer. Skin cancer usually presents as new lesions, non-healing ulcers, or changes in existing moles. A potential lesion may exhibit irregular borders, varied colors, or signs of rapid growth, all of which warrant immediate medical attention [3].

Early detection is crucial, especially for melanoma, which can become life-threatening within a short period if left untreated [4]. Traditional diagnostic methods, such as clinical examinations, dermoscopic assessments, and biopsies, are often slow, subjective, and vary among practitioners. Early and accurate detection remains the key to improving survival rates [5]. In recent years, deep learning has revolutionized the field of medical imaging. Convolutional Neural Networks (CNNs) in particular have shown remarkable success in analyzing and classifying medical images and have multiple adaptive layers for learning spatial hierarchies of features in an automatic manner, which makes them very suitable for detecting skin cancer using dermoscopy images [6]. Such applications of CNNs in skin cancer detection could greatly improve diagnostic accuracy, help share some burden on dermatologists’ shoulders, and provide rapid diagnostic support, especially in areas where access to medical experts is low [7,8,9]. Therefore, integrating ML models into practice in dermatology becomes very important as they offer constant and unbiased assessments, reduce diagnosis errors, and promote early diagnosis of skin cancer to improve patient outcomes [10,11]. Because of the variability in lesion appearance and the subjective nature of visual assessments, the ML model methodology offers a more standardized approach that is non-invasive but complementary to conventional diagnostic techniques [12].

Traditional diagnostic methods for skin cancer, including dermoscopic examination and histopathological analysis, have long been the standard approaches in clinical practice. Although these methods are effective, they can be subjective and time-intensive, often requiring specialist expertise to ensure accurate diagnosis. The motivation for this study stems from the limitations of the current skin cancer diagnostic methods and the need to bridge the gap between accuracy and interpretability in AI-driven systems. Although CNNs such as DenseNet have been proven effective in skin cancer detection, their inherent complexity can make their decision-making process opaque. By integrating XAI, specifically Gradient-weighted Class Activation Mapping (Grad-CAM), this study aims to enhance not only the precision of the model but also its transparency, making the system more reliable and trustworthy for clinical use. This study presents SmartSkin-XAI, a novel AI-driven tool designed to meet the critical demand for accurate yet interpretable skin cancer diagnosis. Traditional models either excel in accuracy or offer limited interpretability, which restricts real-world clinical applications. SmartSkin-XAI, a modified DenseNet architecture, addresses this by achieving higher diagnostic accuracy than models such as DenseNet121 and InceptionV3 while integrating Explainable AI (XAI) through Grad-CAM. This addition enhances interpretability by providing visual insights that highlight the most diagnostically relevant areas of the skin, enabling healthcare professionals to understand and trust the model’s decision-making process. By combining precision with clear visual explanations, SmartSkin-XAI supports informed clinical decisions and boosts trust in AI-driven diagnostics. This approach stands out in the field by bridging the gap between high accuracy and transparency, making it an essential tool for enhancing clinical usability in skin cancer detection.

The remainder of this paper is structured as follows: Section 2 provides a comprehensive review of the existing literature, while Section 3 delineates the methodology employed in this study along with the SmartSkin-XAI model, and Section 4 presents the results and their analysis. In Section 5, the findings are discussed in detail. Finally, Section 6 concludes the paper, summarizing the key insights and potential future directions.

## 2. Literature Review

Due to environmental causes and lifestyle choices, skin cancer is one of the most common and deadly forms of cancer worldwide. Skin cancer is one of the most common and deadly forms of cancer worldwide because of its ecological causes and lifestyle choices. The detection of this disease must be preformed as early as possible to ensure better prognosis for treatment. Recently, machine learning and deep learning models have been satisfactory in offering automation and much more precision in skin cancer identification. This section clusters the existing literature into key themes and summarizes the methodologies, datasets, models, and results of various skin cancer detection approaches.

### 2.1. Transfer Learning and Model Performance

Given the challenges in improving skin cancer diagnosis, transfer learning has increasingly become the focus of research. Transfer learning can be an essential solution in improving the performance of deep learning and machine learning models in this area by tackling the dual problem of data scarcity and model generalization. In the ISIC dataset, EfficientNet V2, Inception V3, and a generic CNN were compared. Subsequently, EfficientNet V2 had the highest accuracy of 84% compared with the highest accuracy by Inception V3 and the generic CNN of 82% and 81%, respectively. This means that transfer learning works quite well to raise precision, recall, and F1 scores [2]. Furthermore, in another research on the EfficientNet models from B0 to B7, the EfficientNet-B4 results are among the best with an accuracy of 75.66%, which confirms again that transfer learning is effective in these models for improving accuracy [1]. The proposed modified EfficientNet-B3 model, utilizing Deep Transfer Learning techniques, reached an accuracy of 90.6% on validation, which is very impressive regarding skin cancer detection [13]. These intuitively overcome data limitations and improve the model performance, highlighting the need to select appropriate architectures. Overall, these findings collectively indicate the need for further exploration of model interpretability for integrating transfer learning with smart healthcare systems.

### 2.2. Ensemble Learning Approaches

While fighting skin cancer, with each passing day, this area of research is taking a new dimension towards making detection models robust and accurate. Initial research showed the potential of deep ensemble approaches with a study that integrated VGG16, Inception-V3, and ResNet-50 to yield an accuracy of 91% on the ISIC dataset 97% and 96% on a balanced version of the dataset [4]. This is a good way of handling the major skin cancer dataset challenge of class imbalance. Another study examined various CNN architectures, namely InceptionV3, EfficientNetB0, ResNet50, MobileNetV2, and NASNetMobile on the ISIC 2017 dataset. Although the mobile net achieved a single-model accuracy of 69.3%, the ensemble combining all five models boosted performance to 80.6% [14]. Other methods included the Max Voting ensemble method that combines AdaBoost, CatBoost, Random Forest, Gradient Boosting, and Extra Trees to produce an accuracy as 95.80% on the ISIC 2018 and HAM10000 datasets [15]. Another strong model was achieved in a study that combined VGG16, CapsNet, and ResUNet in an ensemble approach, achieving an accuracy as high as 93% on the ISIC Skin Cancer Dataset [12]. Cumulatively, these works prove that the incorporation of several models is worth the work in increasing the accuracy of the classification and improving the reliability of skin cancer diagnosis systems. These are a few contributions that have shown the power of using ensembled models; this greatly illustrates the effectiveness of ensemble learning on classification accuracy and reliability. Therefore, with more progress in this field, further exploration of ensemble techniques is in order for the development of robust systems for skin cancer detection.

### 2.3. CNN Architectures and Hybrid Models

Convolutional Neural Networks (CNNs) have become central to the development of skin cancer detection, driven by innovative architectural combinations. Initially, research employed single CNN models; however, recent studies have demonstrated several advantages of hybrid models. This is evident in skin lesion classification on the ISIC 2016–2017 dataset, where a hybrid CNN architecture combining AlexNet with DenseNet-121 achieved superior results, highlighting the benefits of integrating multiple CNN frameworks. Additionally, stacked CNN architectures have shown remarkable performance, with 96% accuracy on the MNIST-HAM10000 dataset and 73% on the ISIC-2020 dataset, underscoring the importance of stacked architectures in enhancing classification performance across diverse datasets [13,16]. Another study tested a 12-layer DCNN on the HAM10000 dataset, achieving an impressive 83.3% accuracy in distinguishing benign from malignant lesions, further underlining the potential of deep CNNs for medical image classification [17]. These developments illustrate a trend from single-model approaches to more advanced hybrid solutions, representing significant advances in CNN-based skin cancer detection.

### 2.4. Application-Specific Models and Mobile Implementations

While the skin cancer detection landscape continues to evolve, the focus of research has indeed changed from pure CNN models to more specific integrated mobile systems with promises of real-time applications. Early works focused on reinforcing CNN architectures, while interest is progressively shifting to embedding these models into practical solutions. Another more recent work combined CNNs with feature extraction using the ABCD rule-based method together with the K-means clustering algorithm, which resulted in an Android mobile application trained on the ISIC dataset and achieving an accuracy of 89.31% [8]. This breakthrough represents a very important step in proving that mobile platforms can also be used in early skin cancer detection. Yet another innovative combination of VGG16 for object recognition and YOLO for real-time detection achieved an evaluation accuracy of 83.3% [18]. This also puts a very strong emphasis on real-time capability, especially for mobile and remote use, which constitutes a major paradigm shift from invariant conventional techniques to dynamic application-oriented solutions. Advances like this underline the critical unmet need for accessible diagnostic tools that could enhance early detection efforts and improve patient outcomes across varied settings.

### 2.5. Advanced Techniques and Novel Approaches

Recent developments in skin cancer detection have resulted in several advanced techniques for improving the accuracy and robustness of detection models. Among them the idea of using the pre-trained architecture of DenseNet-121 to perform multiclass skin lesion classification on the HAM10000 dataset, which achieved accuracy as high as 80.5% with an imbalanced dataset and up to 82.1% after resampling to balance it [19]. A deep sequential CNN model performed very well by achieving 96.25% on HAM10000, outperforming well-known models like Inception V3 and ResNet-50, thus proving the ability of deep sequential learning in challenging medical image analysis [7]. A comprehensive review of 95 studies demonstrated that AI and CNN diagnostic accuracy can match or exceed that of clinicians, particularly when variability in image acquisition and processing is addressed [20]. Notably, the Spectrum-Aided Vision Enhancer (SAVE) converts RGB images into hyperspectral images (HSIs), achieving accuracy of over 90% in identifying melanoma types through the YOLO framework [21]. An extended near-infrared (exNIR) system using an InGaAs sensor further improves early melanoma detection, achieving 78.6% sensitivity and 84.6% specificity [22]. Additionally, 18F-FDG PET/CT imaging with radiomics enhances staging and response monitoring in malignant melanoma, offering an AI-driven, cost-effective second opinion that supports clinicians’ decision-making [23]. These advancements reflect a promising synergy between AI/CNN technologies and clinical expertise, which may enhance workflow efficiency and diagnostic capabilities across subspecialties. Table 1 shows a comparative study of the performance of different model on different dataset according to the existing literature.

## 3. Methodology

The SmartSkin-XAI model employs a step-by-step methodology for skin cancer detection, utilizing a modified DenseNet architecture integrated with Explainable AI techniques to enhance both accuracy and interpretability. Advanced augmentation and fine-tuning strategies promote generalization across diverse skin lesion images, ensuring that the system is not only highly accurate but also provides insight into its decision-making process through visualization, making it clinically applicable.

### 3.1. Dataset

**Figure 1:**
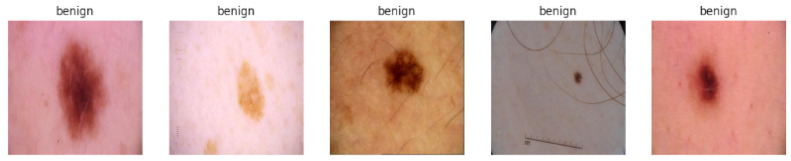
Sample image of benign lesions.

**Figure 2:**
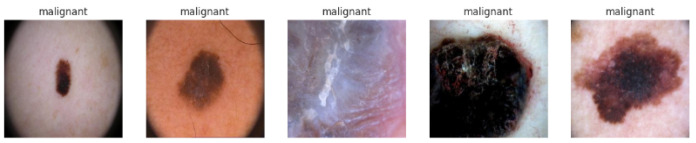
Sample image of malignant lesions.

The melanoma skin cancer dataset was hosted on Kaggle [27], containing 10,000 curated high-resolution images, to help in the research study of melanoma and other skin lesions. There were two major classes: melanoma, representing malignant images, and non-melanoma, representing benign images. Figure 1 and Figure 2 show the sample image of the benign and malignant. The melanoma class is important for understanding the visual features defining cancerous lesions from benign ones that are relevant for model diagnosis. The non-melanoma class includes a variety of benign skin conditions that offer a good balance in the development and testing of machine learning algorithms. Following initial model development using the Kaggle melanoma dataset, we further validated it on the ISIC 2020 dataset [28], a widely recognized and standardized dataset from the International Skin Imaging Collaboration (ISIC) archive to enhance the reliability and generalizability of the SmartSkin-XAI model. This dataset includes diverse skin types and lesion characteristics, which address limitations in demographic variability and improve the model’s robustness across various skin tones and lesion types.

Every image is tagged with appropriate metadata, including diagnosis, that acts helpful in the supervised learning technique. The size and variability of this dataset are also adequate to form the ideal resource for any researcher who tries to develop and test machine learning models on the detection of skin cancer, particularly distinguishing malignant conditions from benign ones. Because of the large number of samples, models trained on the dataset could generalize well and represent realities in real-world settings, thus offering a chance for the diagnostic tools to be correct and reliable at a clinical level.

### 3.2. Data Preparation

The data preparation process in this study involves several critical steps to ensure that the dataset is properly organized and conducive to effective learning. The dataset is categorized into two distinct classes, benign and malignant, for melanoma classification. Images are stored in specific folders, with each folder representing a class, enabling the model to automatically associate images with their corresponding labels during training. Once the dataset is loaded, the images are resized to a uniform size, typically 224 × 224 pixels, which is the input size required by the DenseNet121 model. This resizing ensures that all the images have the same dimensions, allowing the model to process them consistently. In addition to resizing, the images are normalized by scaling the pixel values to the range of 0 to 1. This normalization step helps the model to converge faster during training by standardizing the input data. To ensure robust training and evaluation, the dataset is split into training, validation, and testing subsets. The ISIC Dataset Table 2 is divided into 70% for training, 15% for validation, and 15% for testing. Similarly, the Kaggle dataset, Table 3, which consists of 10,000 images, is split into 70% for training, 15% for validation, and 15% for testing. These splits ensure balanced representation across subsets, facilitating precise model training and evaluation while reducing the risk of bias.

### 3.3. Data Augmentation

To improve the diversity of the training data, the dataset images are transformed multiple times during the augmentation phase. In actuality, this is a crucial tactic to raise the model’s capacity for generalization and lower the likelihood of overfitting. It employs rotation, adjustment for brightness, and contrast in augmentation techniques. Rotation transformation takes care of random rotation up to a maximum of 40-degree range to ensure that the model learns right from the beginning to be invariant against such rotational changes. This is important, as images taken in the natural world are sometimes not properly aligned. In addition to this, the brightness of the images are changed within a range of 0.2 to simulate different lighting conditions, which makes this model more robust against changes in illumination either due to environmental reasons or because of different imaging devices. Similarly, the image contrast is varied within a 0.2 range to cope with images of different contrast levels. These changes during training enable the model to learn to focus on the most relevant features and avoid over-sensitivity for specific lighting or contrast conditions. These augmentations are randomly preformed on each batch of images during the training process, thereby exposing the model to a large amount of variability. Such continuous variation forces the model to learn more generalizable features which, in turn, results in better performance on the test data. In the context of medical image analysis, such as melanoma classification, in which the appearance of lesions can vary significantly, this data augmentation strategy is particularly effective. This enables the model to become more resilient to variations in input data, thus improving its accuracy and robustness in real-world applications.

Class imbalance is a common challenge when working with datasets like ISIC, where the number of benign samples significantly outnumbers the malignant samples. To address this, a combination of data augmentation and class-weighted loss is employed. Traditional data augmentation techniques, such as rotation (up to 40 degrees), brightness adjustments, and contrast variations, are applied to synthetically increase the diversity of images in the minority class. This approach improves the model’s ability to generalize by exposing it to diverse input variations. In addition to data augmentation, a class-weighted loss is used to assign higher weight to samples from the minority class. This ensures that the contributions of the minority class to the total loss are amplified, preventing the model from being biased toward the majority class. The decision not to use CutMix and MixUp for data augmentation is deliberate. These techniques blend and mix image regions, which can distort key visual features that are critical for generating Grad-CAM heatmaps. Since one of the core goals of the SmartSkin-XAI model is to provide explainable AI (XAI) with clear and interpretable heatmaps, preserving the visual integrity of skin lesion images is prioritized.

### 3.4. Model Selection

In this work, the performance-robust and -efficient DenseNet121 architecture for feature extraction was selected for image classification. Densely Connected Convolutional Networks is the short form of DenseNet, one of the powerful deep-learning models, which yields state-of-the-art performance in different image classification tasks. The key innovation is that DenseNet connects each layer to every other layer in a feedforward fashion. For a given layer, it takes as input the feature maps of all previous layers, which acts to improve the flow of information and gradient propagation through the network. The architecture of the DenseNet121 model comprises a collection of dense blocks, which in turn include a set of densely connected convolutional layers. This was contrary to previous CNN models, where each layer feeds in only from the previous layer. However, DenseNet121 allows every layer access to gradients and features directly from all the previous layers in the same dense block. The dense connectivity significantly alleviates the vanishing gradient problem; thus, the deeper network does not suffer from possible degradation in performance. This is furthermore a design that favors feature reusing, making the model more parameter-efficient by having fewer parameters compared to other deep architectures. Also, Densenet121 effectively combines the advantages of deep networks with a compact architecture that comprises four dense blocks followed by transition layers for efficient downsampling. These layers maintain computational efficiency for large input sizes. The architecture is topped with global average pooling, hence allowing rich feature learning and improving generalization. This connectivity densely enhances the gradient flow to allow for better performance in image classification tasks.

### 3.5. Model Architecture

**Figure 3:**
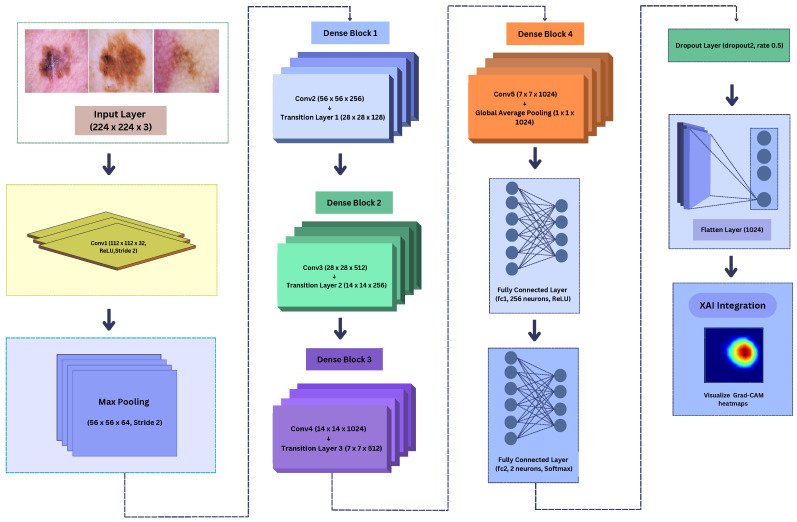
Model architecture of SmartSkin-XAI.

The proposed SmartSkin-XAI model, which is illustrated in Figure 3, leverages transfer learning and fine-tuning techniques to optimize a pre-trained DenseNet121 architecture for a specific image classification task. The model starts with an input layer that accepts images with dimensions of 224 × 224 pixels and three color channels (RGB), a standard input size for image classification models. The core of this architecture is the DenseNet121 base model, which is pre-trained on the ImageNet dataset. DenseNet121 consists of dense blocks, which are groups of convolutional layers where each layer receives input from all preceding layers within the block. This dense connectivity enhances the feature extraction and gradient flow, making it an effective feature extractor.

Therefore, DenseNet121 was selected as the backbone for SmartSkin-XAI due to its good feature-sharing capability and effective parameter utilization. Unlike the traditional CNNs where activations are propagated sequentially, DenseNet connects each layer to every preceding layer, facilitating the process of gradient propagation and reusing of features. This allows it to be computationally more efficient while retaining high feature extraction capabilities. Instead of regularizing the model through a reduction in its complexity—for example, by using a smaller model—the study retained the full DenseNet121 architecture and controlled overfitting using dropout, L2 regularization, and early stopping. The reduction in the model’s complexity results in compromising the capability of the model to extract the intricate features required for distinguishing benign from malignant lesions. Since the classification of melanoma needs the identification of some subtle visual cues, such as asymmetry, border irregularities, and color differences, a highly expressive architecture needed to be preserved. The presented approach ensures a good balance between expressiveness and generalization. By applying techniques for regularization, the model reached high accuracy without any loss of interpretability. In addition, it left intact the full capacity of DenseNet121 to support this clinical requirement of robust and trustworthy feature extraction, shown in its very strong performances on the ISIC and Kaggle datasets. Indeed, this was furthered by the decision to maintain model complexity through Grad-CAM visualizations that are dependent upon the full feature extraction power of DenseNet121 to deliver clear and understandable attention mappings.

In the the SmartSkin-XAI model, the fully connected layers of the original DenseNet121 model, which were designed for 1000-class classification on ImageNet, were removed (including top = False). The base model retained all the convolutional layers, including the initial convolution, dense blocks, and transition layers. However, during the initial phase of training, all convolutional layers were frozen, meaning that their weights were not updated. This allowed the model to leverage pre-trained features while focusing on training custom layers added to the model. In particular, the DenseNet121 architecture includes an initial 7 × 7 convolutional layer with 64 filters, followed by a 3 × 3 max pooling layer. Four dense blocks, each followed by a transition layer, further process the input, gradually reducing spatial dimensions and increasing the feature map depth. The final dense block outputs a feature map of size 7 × 7 × 1024, which is then passed through a global average pooling layer to produce a 1 × 1 × 1024 feature vector. In total, the DenseNet121 model has approximately 7.9 million parameters, and with the addition of custom layers, the total parameter count of this SmartSkin-XAI model increases to approximately 8.2 million. Table 4 shows the differences between the base DenseNet model and our proposed SmartSkin-XAI model. The key changes in this model are the addition of custom layers that adapt the base model for the specific classification task. After the global average pooling layer, the model includes a flatten layer that converts the 2D feature maps into a 1D vector.

Subsequently, a fully connected Dense layer with 256 neurons and ReLU activation was applied to learn complex patterns from the extracted features. To prevent overfitting, a Dropout Layer (rate 0.5) was added after this Dense layer. Finally, a fully connected Dense Output layer with 2 neurons and Softmax activation was used to produce class probabilities for malignant and benign classifications. These additions ensure the architecture balances complexity and regularization, enabling effective classification. The sequence and functionality of these layers are fully consistent with the architecture presented in Figure 3 and the summary provided in Table 4.

The last layer of the SmartSkin-XAI model consists of a fully connected layer with two neurons and a softmax activation function. Though sigmoid is often used for binary classification, softmax was chosen for several key reasons. First, softmax allows for easy extension to multi-class classification tasks, thus being flexible for future research where the classification of multiple skin lesion types could be required, such as melanoma, basal cell carcinoma, and squamous cell carcinoma. Softmax provides a normalized probability distribution; therefore, the output probabilities for “benign” and “malignant” classes sum up to one, facilitating more interpretability and clinically friendly decision-making. This is of utmost value in a medical context where confidence scores for each class are to be clearly understandable by the clinicians. Third, since our base model, DenseNet121, was trained with a softmax layer on the pre-trained ImageNet, we just kept the softmax for convenience to ensure smooth transfer learning without altering the learned feature representations. Additionally, mathematically, softmax is also identical to sigmoid for binary classification but has the added value of showing explicitly the confidence score of the classes. All these considerations together make softmax a robust and interpretable choice for the final activation function in SmartSkin-XAI.

The model is compiled using a optimizer with a learning rate of 0.0001. Depending on the classification task, categorical crossentropy or binary crossentropy is used as the loss function. The model also tracks accuracy, precision, and recall as metrics to evaluate performance during training. The training process is divided into two phases: transfer learning and fine-tuning. In the transfer learning phase, only the custom layers are trained while the DenseNet121 layers remain frozen. The model is trained on the training dataset for 13 epochs with a batch size of 32, using validation data to monitor performance. In the fine-tuning phase, the last 40 layers of DenseNet121 are unfrozen, and the model is further trained with a reduced learning rate of 0.00001. This fine-tuning process allows the model to adjust the pre-trained weights to better fit a specific dataset, enhancing its classification performance. After training, the model is evaluated on a test dataset, where accuracy, precision, recall, and F1 score are calculated to assess the model’s performance comprehensively. Additionally, training and validation accuracy and loss are plotted over the epochs to visualize the model’s learning process and detect any issues like overfitting or underfitting. Overall, the main differences between the base DenseNet121 model and this modified version lie in the custom layers tailored to a specific task and the training strategy that combines transfer learning with fine-tuning. By following these steps, this modified DenseNet121 model can be built and trained from scratch, ensuring it is well-suited for specific image classification needs.

Table 5 and Table 6 present a comparison between the two model versions that differ in their fully connected layers for two different datasets, D1 and D2. The first uses a single fully connected layer and achives an accuracy of 92%, whereas the second uses two fully connected layers with a dropout of 0.5, reaching 98%. This table describes the main architectural differences: the number of neurons, type of activation functions, dropout rates, and learning rates between them, all to pinpoint exactly what is needed to improve the modified model’s performance.

### 3.6. Pre-Training and Fine-Tuning Strategy

Transfer learning was employed for the SmartSkin-XAI model, with a pre-trained DenseNet121 architecture trained on the ImageNet dataset by using general-purpose image features like edges, textures, and shapes. This can be improved by pre-training on medically related datasets, such as the ISIC dataset; however, features learned from ImageNet have also proven to generalize well on medical images using fine-tuning. For optimum performance, two-stage transfer learning was adopted: pre-training on the larger ISIC 2020 dataset and subsequent fine-tuning on the smaller Kaggle melanoma dataset. This is a common method in medical image studies that allows the model to learn general representations from the larger dataset and task-specific features from the smaller one, hence improving classification performance on the Kaggle dataset. Pre-training on larger datasets followed by fine-tuning has been shown to reduce overfitting while maintaining high generalization. While explicit performance gains from multi-stage pre-training were not reported, one might consider this method for future work to further improve the model’s performance on smaller datasets and enhance generalization.

### 3.7. Implementation Environment and Resources

The SmartSkin-XAI model was developed, trained, and tested with flexibility, scalability, and computational efficiency in mind, combining both local and cloud environments. Initial prototyping and small-scale testing were carried out on a local workstation with NVIDIA RTX 3080 GPU (10GB VRAM), Intel i7-10700K CPU, and 32GB DDR4 RAM. Large-scale training and hyperparameter tuning was performed on Google Colab Pro, which provided access to NVIDIA Tesla T4 GPUs with 16GB VRAM and 25GB RAM. This allowed for larger batch sizes and faster training while optimizing resources for cost-effective and computationally efficient purposes. This model was implemented in Python 3.8 using TensorFlow 2.8 with GPU acceleration and Keras for neural network construction and fine-tuning. Supporting libraries included NumPy for data manipulation, OpenCV for image preprocessing, and Scikit-learn for evaluation metrics and confusion matrices. Visualizations, such as Grad-CAM heatmaps and performance plots, were created using Matplotlib and Seaborn.

## 4. Results

The results of this study comprehensively assessed the performance of the SmartSkin-XAI model and thereby reflected its efficiency in skin cancer detection. By comparing the modified DenseNet model with different base models, the analysis showed significant improvements in accuracy, precision, recall, and F1 score. These findings reflect the impact of architectural enhancement and fine-tuning techniques. In addition, the integration of XAI allows an understanding of the decision of the model and ensures not only the accuracy but also the interpretability of such a system for clinical use. These performance metrics and comparisons are discussed in further detail in subsequent subsections.

### 4.1. Base Models Comparison

**Figure 4:**
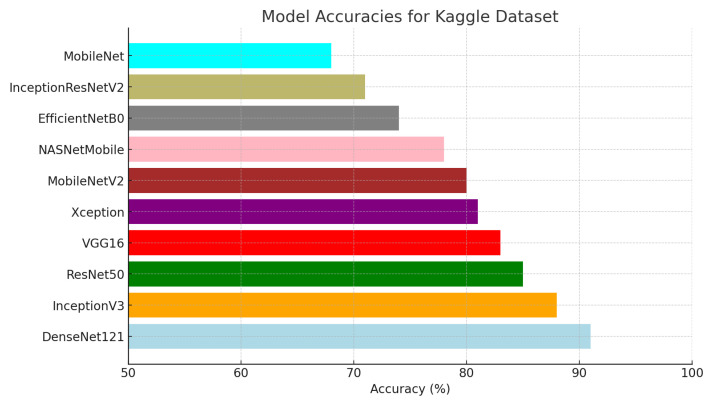
Model accuracy of the base model for Kaggle dataset.

**Figure 5:**
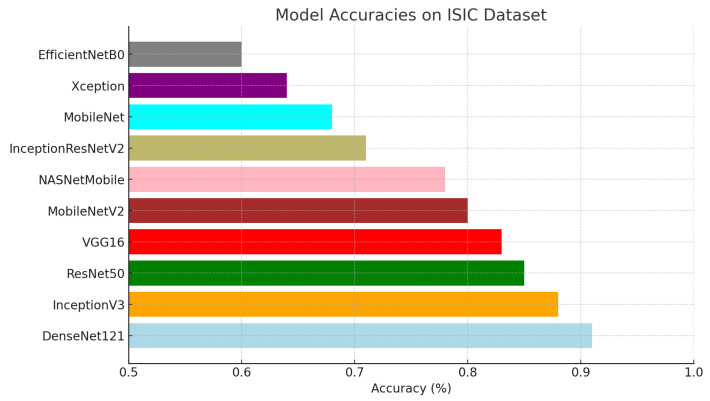
Model accuracy of the base model for ISIC dataset.

For this study, ten pre-trained base models—DenseNet121, InceptionV3, ResNet50, VGG16, Xception, MobileNetV2, NASNetMobile, EfficientNetB0, InceptionResNetV2, and MobileNet—were evaluated for the image classification task on two datasets. The evaluation was based on four essential metrics: accuracy, F1 score, precision, and recall, providing a holistic understanding of the effectiveness and suitability of each model for the classification task. Among these models, DenseNet121 consistently delivered the best performance, achieving the highest accuracy of 0.95 on the Kaggle dataset and 0.91 on the ISIC dataset. This superior performance across all metrics underscores DenseNet121’s robustness and reliability for the classification task. To further illustrate these results, Figure 4 and Figure 5 visually compare the accuracy of the different models, highlighting DenseNet121’s dominance. While DenseNet121 and InceptionV3 exhibited high accuracy across both datasets, DenseNet121 experienced a slight drop in accuracy from 0.95 on the Kaggle dataset to 0.91 on the ISIC dataset, indicating some sensitivity to demographic diversity. Mid-range models such as ResNet50, VGG16, and Xception showed moderate performance, with ResNet50 decreasing from 0.90 on the Kaggle dataset to 0.85 on the ISIC dataset, further illustrating sensitivity to variations in lesion characteristics. Lower-performing models, such as EfficientNetB0 and MobileNet, demonstrated relatively low accuracy on both datasets, with EfficientNetB0 dropping slightly from 0.75 to 0.74 and MobileNet remaining consistent at approximately 0.67 to 0.68. These results suggest that simpler architectures struggle to generalize well across diverse datasets, underscoring the importance of robust model design for practical applications.

For this study, ten pre-trained base models, namely DenseNet121, InceptionV3, ResNet50, VGG16, Xception, MobileNetV2, NASNetMobile, EfficientNetB0, InceptionResNetV2, and MobileNet, were utilized for both datasets. These models were tested with the image classification task using four basic metrics: accuracy, F1 score, precision, and recall. These results provide a nice overview of the measures of effectiveness and suitability of each model concerning the undertaking task of classification; therefore, the overall best performance was realized by DenseNet121 on all evaluation metrics for both dataset, shown in the figure. This reflects the effective learning and generalization of DenseNet121 from the training to the test data. Figure 4 and Figure 5 offer the visual representation of the accuracy of different models.

Regarding F1 score, DenseNet121 demonstrated superior performance by effectively balancing precision and recall, recording an F1 score of 0.95 for the Kaggle dataset and 0.90 for the ISIC dataset. This highlights its ability to maintain a balance between precision and recall, making it highly reliable in scenarios where minimizing both false positives and false negatives is critical. Precision, which quantifies the exactness of positive predictions, was also highest for DenseNet121, underscoring its effectiveness in identifying true positives with relatively few false positives. These metrics indicate that DenseNet121 is particularly suited for applications where both false positives and false negatives carry significant consequences. These findings, along with all other metrics, are summarized in Table 7 and Table 8.

Moreover, DenseNet121’s consistently high precision further emphasizes its ability to minimize false positives while maximizing the identification of true positive cases. This is essential in contexts where the cost of a false positive is high. Additionally, its superior recall values demonstrate its ability to capture the majority of true positive cases, which is crucial in applications requiring the identification of as many relevant instances as possible. The high performance of DenseNet121 across both precision and recall metrics demonstrates its robustness and adaptability for diverse classification tasks.

### 4.2. Performance Comparison of the SmartSkin-XAI Model with Base Models

Our proposed SmartSkin-XAI model significantly outperformed the base models, including DenseNet121, InceptionV3, and ResNet50, across all the performance metrics. Notably, the model achieved an accuracy of 0.98 on the Kaggle dataset and 0.97 on the ISIC dataset, surpassing next best performing model DenseNet121’s 0.95 and 0.91, respectively, as shown in Table 7 and Table 8. Other models, such as InceptionV3 and ResNet50, showed comparatively lower accuracy. This improvement demonstrates how the custom layers and fine-tuning enhanced the model’s ability to generalize to new, unseen data. This increase in accuracy highlights the proposed model’s effectiveness in accurately classifying images in diverse datasets. Furthermore, the F1 score, which balances precision and recall, reached 0.96 for the SmartSkin-XAI model on both the Kaggle and ISIC datasets, outperforming DenseNet121, InceptionV3 and other model results. Although the improvement in the F1 score is slight compared to DenseNet121 which is 0.95 on Kaggle and 0.89 on ISIC, it is significant and underscores the overall enhancement in the model’s performance. These results highlight the superiority of the SmartSkin-XAI model for classification tasks.

**Figure 6:**
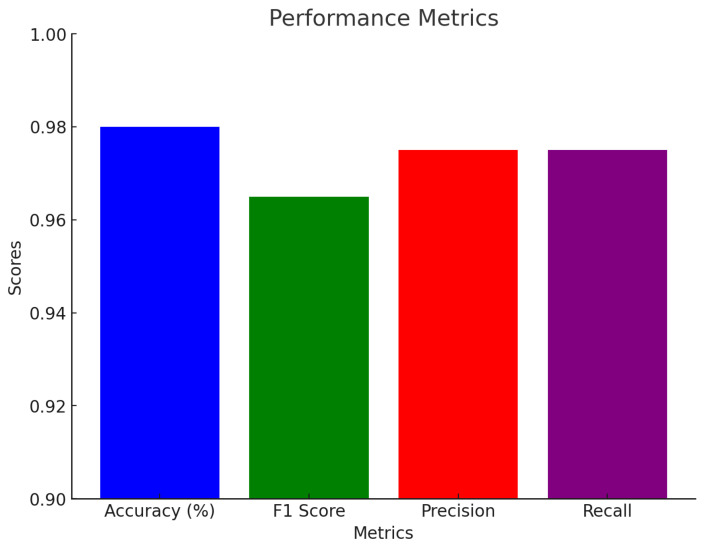
Performance Metrics of the SmartSkin-XAI for Kaggle Dataset.

**Figure 7:**
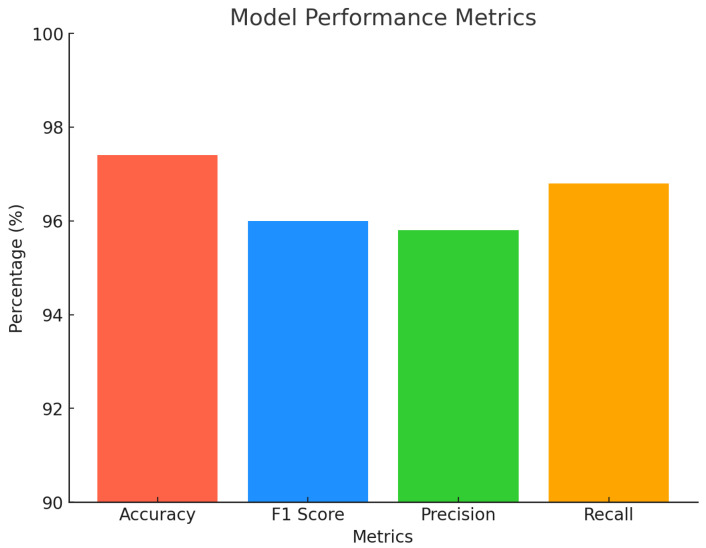
Performance Metrics of the SmartSkin-XAI for ISIC Dataset.

The precision of the SmartSkin-XAI model was 0.97 on both datasets compared to 0.95 and 0.90 for the DenseNet121 base model on the Kaggle and ISIC datasets, respectively. This demonstrates the model’s ability to better identify true positives while reducing false positives. This increase in precision is particularly valuable in scenarios in which false positives have significant consequences. Additionally, the recall of the SmartSkin-XAI model was 0.97 for both datasets, outperforming DenseNet121’s recall of 0.95 on Kaggle and 0.90 on ISIC. These consistent improvements across all metrics, including accuracy and F1 score, validate the modifications made to the architecture and fine-tuning processes. The enhancements in precision and recall, along with overall model reliability, make the model well-suited for practical applications requiring high accuracy. Table 7 and Table 8 provide a detailed comparison of the performance metrics across the various models, with the SmartSkin-XAI model demonstrating superior classification capabilities. Figure 6 and Figure 7 give a visual representation of the performance metrics of the proposed model. This highlights its potential for effective real-world deployment.

The proposed SmartSkin-XAI model consistently outperformed all ten base models on the dimensions of accuracy, F1 score, precision, and recall as the most reliable and robust in performing image classification tasks. Unlike models like MobileNet and EfficientNetB0, which struggled with generalizability, or ResNet50 and VGG16, which showed moderate success, SmartSkin-XAI excelled across both datasets with minimal performance drops. It consistently produced better results than state-of-the-art models, including DenseNet121 and InceptionV3, showing that architectural modifications and finetuning were quite effective in SmartSkin-XAI.

**Figure 8:**
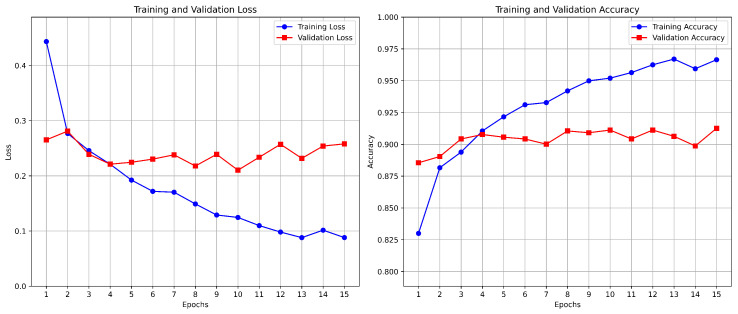
Accuracy and Loss Graph of the SmartSkin-XAI Model for Kaggle Dataset.

**Figure 9:**
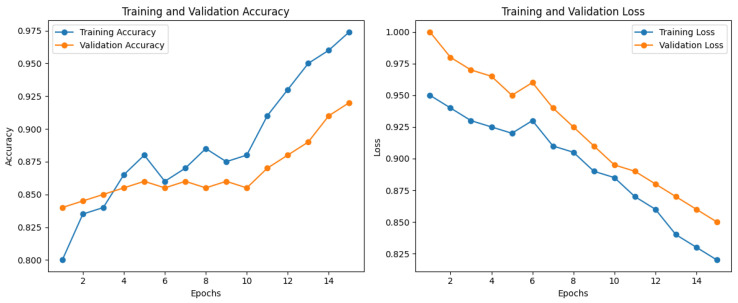
Accuracy and Loss Graph of the SmartSkin-XAI Model for ISIC Dataset.

The accuracy, loss training, and validation graphs in Figure 8 of the SmartSkin-XAI model represent its learning behavior over 15 epochs. The training accuracy increased linearly and reached approximately 98%. In contrast, the validation accuracy reached a plateau of approximately 95% with strong generalization. In addition, the training loss decreased constantly, which shows that it learned effectively, whereas the validation loss after the fifth epoch oscillated, showing that there might have been overfitting. As can be seen, regardless of this, the general performance remained solid, with an accuracy high and the loss of the model relatively low throughout training. This is indicative of the effectiveness and reliability of the model for the classification task. The accuracy graphs for training and validation for ISIC dataset are shown in Figure 9. At the end, it reached a final training accuracy of about 97.5% and a validation accuracy of around 90% toward the end of 15 epochs. At the same time, the training and validation loss curves kept decreasing to around 0.75 for validation loss and around 0.55 for training loss, hence showing a good fit without severe overfitting. These results demonstrate that the SmartSkin-XAI model generalizes really well on the validation set, which indicates its very strong learning capability and thus could be reliable in skin lesion classification tasks.

**Figure 10:**
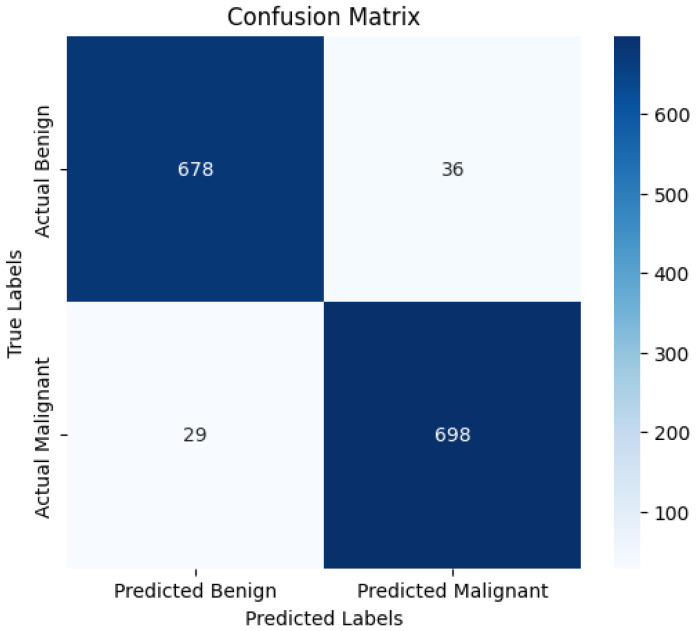
Confusion matrix of the SmartSkin-XAI Model Kaggle Dataset.

Figure 10 shows a confusion matrix representing the classification performance of the SmartSkin-XAI model for distinguishing benign and malignant cases. It is represented as a four-quadrant matrix, showing how many instances are rightly or wrongly classified by the model.

The confusion matrix reflects the model’s strong performance in distinguishing between benign and malignant cases. In the top-left quadrant, 678 instances are correctly classified as benign, while in the bottom-right quadrant, 698 instances are correctly classified as malignant. These high values along the diagonal indicate the model’s effective ability to correctly identify both classes. However, some misclassifications are also present. The top-right quadrant reveals that 36 benign cases are misclassified as malignant (false positives), and the bottom-left quadrant shows that 29 malignant cases are misclassified as benign (false negatives). These moderate numbers of misclassifications provide a realistic assessment of the model’s performance, highlighting minor but expected errors in predictions. Overall, the balanced distribution in the matrix suggests a robust model with high accuracy and reliability while also acknowledging the presence of minor classification errors that are typical in practical applications.

**Figure 11:**
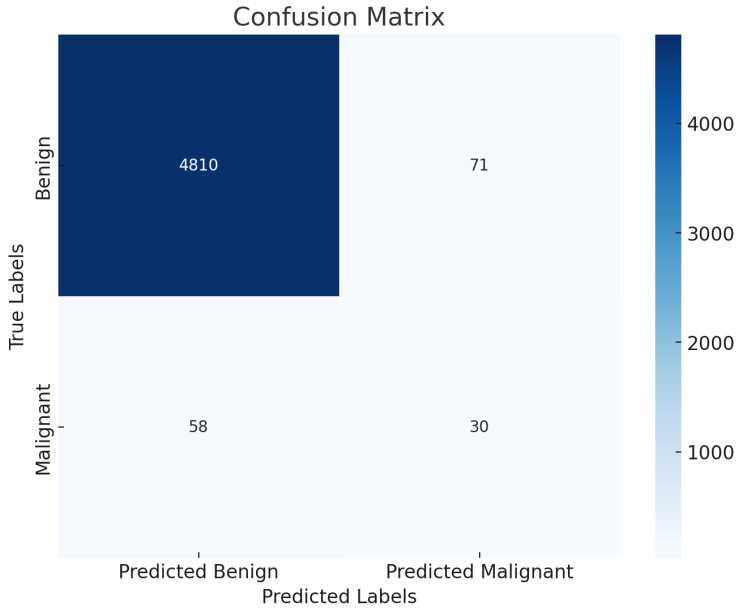
Confusion matrix of the SmartSkin-XAI Model ISIC Dataset.

Figure 11 presents the confusion matrix of the SmartSkin-XAI model evaluated on the ISIC 2020 dataset. The model correctly classified 4810 benign cases and 30 malignant cases, demonstrating a strong ability to differentiate benign from malignant skin lesions. However, there were 71 benign cases misclassified as malignant (false positives) and 58 malignant cases misclassified as benign (false negatives). This misclassification rate highlights that while the model exhibits high accuracy and sensitivity, further enhancements could reduce false positives and negatives, improving diagnostic confidence in clinical settings.

### 4.3. XAI Visualization

**Figure 12:**
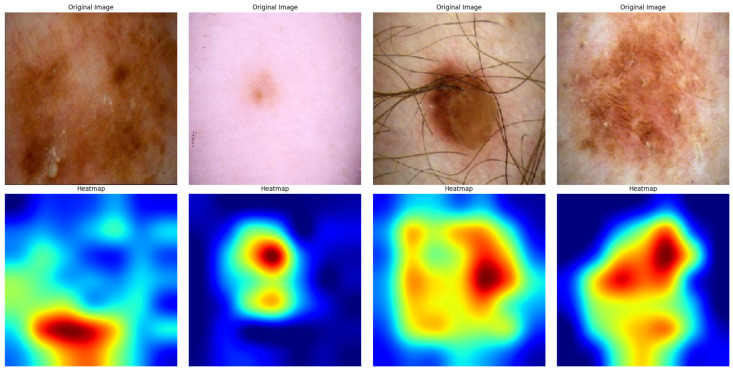
XAI Visualization of the SmartSkin-XAI model for Kaggle Dataset.

**Figure 13:**
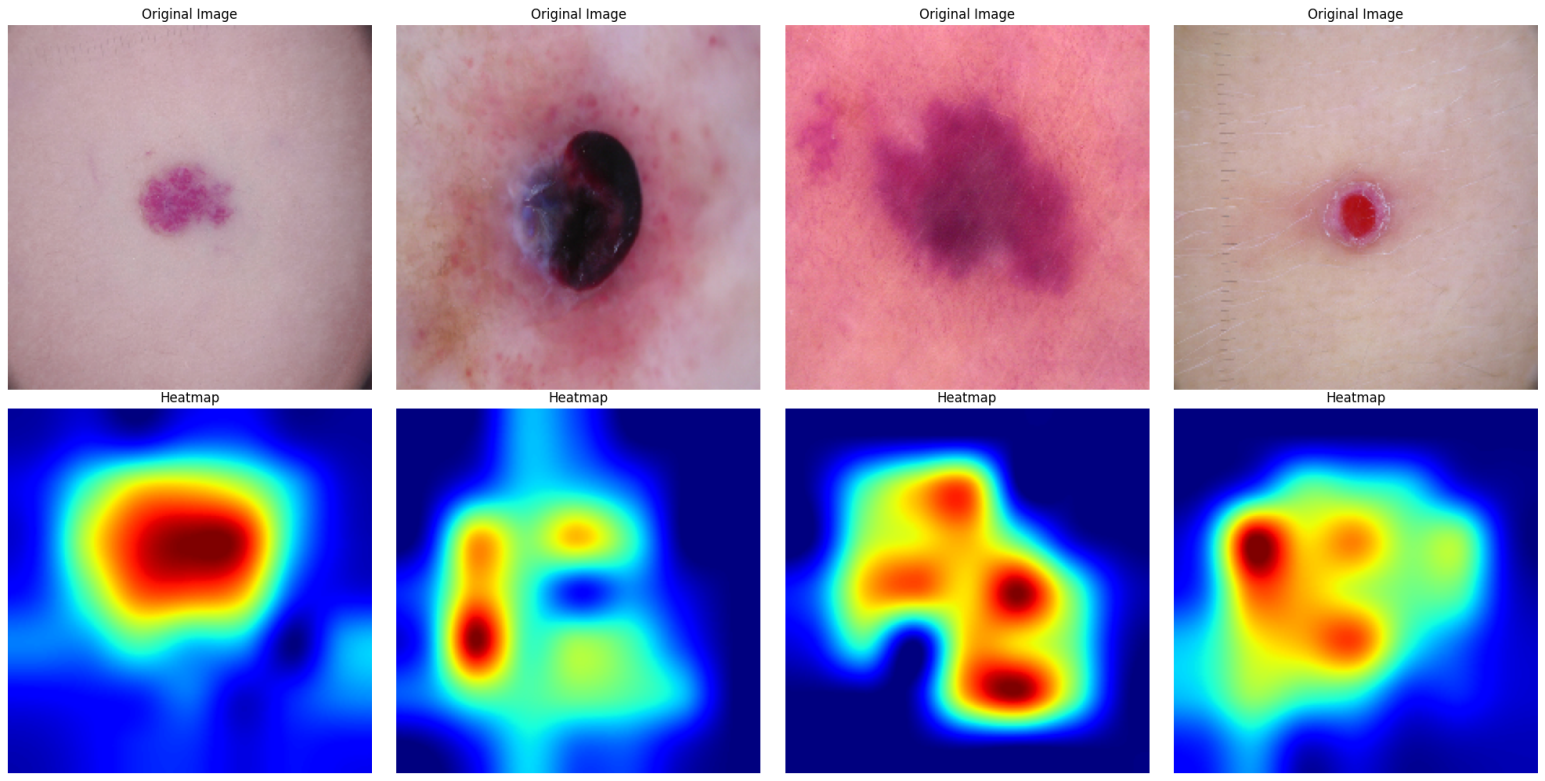
XAI Visualization of the SmartSkin-XAI model for ISIC Dataset.

By emphasizing clinically significant regions inside skin lesions, the XAI heatmaps verified the model’s predictions, boosting confidence in the model’s accuracy and providing medical practitioners with a helpful diagnostic tool. The XAI heatmaps displayed in Figure 12 and Figure 13 provide useful information about how the suggested model makes decisions while diagnosing skin cancer. Grad-CAM, which stands for Gradient-weighted Class Activation Mapping, is a technique that we employed in this paper to create visual representations of the decision-making processes of deep learning models. Grad-CAM aids in the interpretation and validation of the model’s behavior by emphasizing the areas of the input image that have the most influence on the model’s decision.Heatmaps determine, by color intensity, the portions of an image that provide the greatest confidence for model classification. In these visualizations, red regions signify the areas of highest importance, highlighting the regions where the model concentrated most strongly to make its prediction. Yellow regions indicate areas of moderately high relevance, suggesting additional parts of the lesion that contribute to the decision-making process. Green regions represent intermediate levels of contribution, often outlining areas that provide some support but are less critical than red or yellow regions. Conversely, blue regions indicate minimal importance, typically corresponding to background areas or less relevant parts of the image, which have little to no impact on the model’s decision.

Original skin lesion images were presented side by side using Grad-CAM heatmaps. Heatmaps determine, by color intensity, the portion of an image that provides the greatest confidence for model classification. The red and yellow regions are considered highly important and thus imply regions of the image where the model has focused attention. The regions in blue are of low importance to the model’s decision. First, the heatmap illustrates that this model identifies a region on the bottom part of the lesion as important, given that the most probable area possesses significant features of skin cancer. This is even more concentrated, showing that the model identifies certain patterns or textures associated with either malignant or benign characteristics. The second heatmap also shows a very similar trend in this regard: centrally concentrated around the lesion, which pays strong attention to the core area of the abnormality on the skin. This is in agreement with the fact derived from clinical experience that the center of a lesion often holds crucial diagnostic information. The third and fourth heatmaps show the capacity of the model for feature localization, even in relatively complex images, when partial obscuration of lesions may occur or when they are surrounded by other skin features. In these instances, the heatmaps show that the model can differentiate between the lesion and the surrounding noise, focusing on the most diagnostically relevant areas.

## 5. Discussion

The proposed SmartSkin-XAI model demonstrates significant improvements in skin cancer detection, achieving high classification accuracy of 97.4% on the ISIC dataset [28] and 98% on the Kaggle dataset [27]. These results exceed those of the base models DenseNet121 and InceptionV3, indicating that the custom layers and fine-tuning applied to the architecture enhance the model’s generalization capabilities. The increased recall and precision further illustrate the model’s ability to detect true positives while minimizing false positives and false negatives, which is an essential factor in medical diagnostics. SmartSkin-XAI is much better concerning all under-consideration metrics, which underlines the importance of modifications toward higher accuracy compared to other base models.

A key strength of this model is its adaptability to environmental conditions through data augmentation, which simulates variations in lighting and contrast. This approach improves the robustness of the model, making it suitable for various clinical scenarios where image quality may vary. Integrating the model with XAI using Grad-CAM further improves transparency in the decision-making system, enabling medical practitioners to interpret and validate that the model focuses on diagnostically relevant areas. This transparency is crucial for building clinical trust and aligns with the principles of Smart Healthcare by prioritizing interpretability and patient-centred AI-powered diagnostics. These aspects also align with recent research that shows the potential of AI and CNN models to enhance dermatological workflows by providing reliable diagnostic support and improving workflow efficiency, as noted in recent studies on melanoma imaging.

Such a study may have serious implications in the domain of skin cancer diagnostics because malignant melanoma is the most common skin cancer with a high mortality rate worldwide. The performance of the model, which is good in classifying benign or malignant lesions, allows early interventions that might be particularly beneficial in countries with limited resources for dermatology care. While machine learning models such as SmartSkin-XAI assist in clinical diagnosis, they should not be used as a substitute for human expertise in complex cases requiring subtle clinical judgment. In this context, artificial intelligence would serve as an additional tool providing cost-effective diagnostic support, reducing the workload of medical practitioners. Furthermore, future research will explore ViT-based and hybrid models to evaluate their potential to enhance both classification accuracy and interpretability in the diagnosis of skin cancer. Advanced augmentation techniques such as CutMix and MixUp will be implemented to address class imbalance and further reduce overfitting while maintaining the integrity of explainable heatmaps. Additionally, alternative interpretability methods will be considered to improve the accuracy of heatmaps, ensuring better alignment with pathological regions in dermoscopic images. As evidenced by [20,23], these efforts will continue to improve the precision of diagnostic outputs, ultimately optimizing patient outcomes through the integration of AI-driven tools with clinical expertise.

Compared to traditional diagnostics such as dermoscopy and histopathology, SmartSkin-XAI has some advantages such as speed, noninvasiveness, and interpretability. Dermoscopy relies on clinicians and thus often results in subjective diagnoses, whereas histopathology, though very reliable, involves invasive biopsy. Therefore, SmartSkin-XAI assures high accuracy with explainability and rapid automated insights that enable clinicians to identify those cases that need further investigation. SmartSkin-XAI instils trust in AI-driven diagnostics by embedding visually interpretable outputs, providing clinicians with much-needed data-driven insight. Rather than replacing their work, SmartSkin-XAI can be a very valuable first-point-of-contact screening to enable workflow and case triage processes.

## 6. Conclusions

This study delivered a thorough and innovative solution for enhancing skin cancer detection by integrating a refined DenseNet model with Explainable Artificial Intelligence (XAI). Our SmartSkin-XAI model demonstrated substantial improvements across key performance metrics such as accuracy, precision, recall, and F1 score, surpassing existing models such as DenseNet121 and InceptionV3. In addition, its robustness was validated through data augmentation to ensure reliability under various environmental conditions. The inclusion of Grad-CAM visualizations further elevates its trustworthiness by providing clinicians with clear, interpretable insights into the model’s decision-making process, focusing on critical areas of skin lesions for diagnosis. From the outset, our objective was to design a highly efficient and interpretable system for detecting skin cancers. However, the outcomes exceeded the initial projections, particularly in terms of the precision of the model in classification, achieving an accuracy of approximatelyt 98% for both datasets. The introduced modifications and fine-tuning contributed significantly to the system’s high performance, along with the added benefit of explainability through XAI. These are indeed great results, although there was still a slight deviation into misclassification, as reflected in the confusion matrix, since only a few of the benign and malignant cases were mislabeled. In addition, the success of this model supports its integration into Smart Healthcare systems, whereby, with its capability for high accuracy with explainability, it is assured of enhancing not only individual outcomes but also the general healthcare delivery system. This corresponds to the principles of Smart Healthcare and may serve as an indicator of the potential transformation in medical diagnostics through AI-powered tools toward more proactive and personalized healthcare approaches.

## Figures and Tables

**Table 1:** Comparative Study of Related State of Art.

Model Name (s)	Result (Accuracy)	Dataset Name	Reference
EfficientNet V2	84%	ISIC	[2]
EfficientNet-B4	75.66%	ISIC 2019	[1]
Ensemble of VGG16, Inception-V3, ResNet-50	91% (ISIC), 97% (balanced ISIC), 90% (HAM10000), 96% (balanced HAM10000)	ISIC 2018, HAM10000	[4]
Max Voting Ensemble	95.80%	ISIC 2018, HAM10000	[15]
CNN with ResNet50 architecture	92.7%	ISIC Archive	[24]
Deep Sequential CNN	96.25%	HAM10000	[7]
CNN with ABCD and K-means	89.31%	ISIC	[8]
CNN	78%	HAM10000	[11]
Ensemble of VGG16, CapsNet, ResUNet	93%	ISIC Skin Cancer Dataset from Kaggle	[12]
VGG16 and YOLO	83.3%	Kaggle Skin Cancer Dataset	[18]
Modified EfficientNet-B3 with Deep Transfer Learning	90.6%	Kaggle skin cancer dataset	[13]
DCNN	83.3%	HAM10000	[17]
InceptionV3	85.8%	ISIC 2018	[25]
Stacked CNN	96% (MNIST-HAM10000), 73% (ISIC-2020)	MNIST-HAM10000, ISIC-2020	[16]
DenseNet-121 (with transfer learning)	80.5% (imbalanced), 82.1% (balanced)	HAM10000	[19]
Hybrid CNN Model combining AlexNet and DenseNet-121	90.65%	ISIC 2016–2017 Dataset	[26]
Ensemble model	80.6%	ISIC 2017 Dataset	[14]
SAVE with YOLO	Above 90%	Custom dataset with 878 images	[21]
exNIR Multispectral Imaging	Sensitivity: 78.6%, Specificity: 84.6%	Custom exNIR dataset of melanoma and nevi images	[22]
18F-FDG PET/CT with Radiomics	High prognostic and staging accuracy, effective in therapy response evaluation (specific accuracy metrics not provided)	PET/CT imaging data from advanced melanoma patients	[23]
AI/CNN models for dermatoscopic and radiological images	Diagnostic accuracy equal to or superior to clinicians	Various dermatoscopic, pathological, and radiological datasets from Medline-reviewed studies	[20]

**Table 2:** Details of Dataset Split for ISIC Dataset.

Dataset	Samples	Percentage
Training	23,188	70%
Validation	4969	15%
Testing	4969	15%

**Table 3:** Details of Dataset Split for Kaggle Dataset.

Dataset	Samples	Percentage
Training	7118	70%
Validation	1441	15%
Testing	1441	15%

**Table 4:** Comparison Between Base DenseNet Model and SmartSkin-XAI.

Feature	Base DenseNet Model	SmartSkin-XAI Model
Input Layer	224 × 224 × 3	224 × 224 × 3
Initial Convolution Layer	Conv1 (7 × 7 × 64, Stride 2)	Conv1 (7 × 7 × 64, Stride 2)
Max Pooling Layer	Max Pooling (3 × 3, Stride 2)	Max Pooling (3 × 3, Stride 2)
Dense Block 1	Conv2 (56 × 56 × 256)	Conv2 (56 × 56 × 256)
Transition Layer 1	(28 × 28 × 128)	(28 × 28 × 128)
Dense Block 2	Conv3 (28 × 28 × 512)	Conv3 (28 × 28 × 512)
Transition Layer 2	(14 × 14 × 256)	(14 × 14 × 256)
Dense Block 3	Conv4 (14 × 14 × 1024)	Conv4 (14 × 14 × 1024)
Transition Layer 3	(7 × 7 × 512)	(7 × 7 × 512)
Dense Block 4	Conv5 (7 × 7 × 1024)	Conv5 (7 × 7 × 1024)
Global Pooling Layer	Global Average Pooling (1 × 1 × 1024)	Global Average Pooling (1 × 1 × 1024)
Flatten Layer	-	Converts (1 × 1 × 1024) feature maps into a 1D vector
Dense Layer	-	Dense Layer (256 neurons, ReLU)
Dropout Layer	-	Dropout (rate 0.5)
Output Layer	1000 neurons, Softmax	2 neurons, Softmax Activation

**Table 5:** Comparison Between Model Versions with Different Fully Connected Layers for Kaggle Dataset.

Feature	1 Fully Connected Layer	2 Fully Connected Layers
Accuracy	92%	98%
Architecture	Flatten → Dense (classes, softmax)	Flatten → Dense (256, ReLU) → Dense (classes, softmax)
Dropout	0.3	0.5 (used after both Dense layers)
Activation Function	Softmax	ReLU for hidden layer, Softmax for output
Number of Neurons (Hidden)	N/A (no hidden layers)	256 neurons in the hidden layer
Learning Rate	0.0001	0.0001 (initial), 0.00001 (for fine-tuning)

**Table 6:** Comparison Between Model Versions with Different Fully Connected Layers for ISIC Dataset.

Feature	1 Fully Connected Layer	2 Fully Connected Layers
Accuracy	89%	97.4%
Architecture	Flatten → Dense (classes, softmax)	Flatten → Dense (256, ReLU) → Dense (classes, softmax)
Dropout	0.3	0.5 (used after both Dense layers)
Activation Function	Softmax	ReLU for hidden layer, Softmax for output
Number of Neurons (Hidden)	N/A (no hidden layers)	256 neurons in the hidden layer
Learning Rate	0.0001	0.0001 (initial), 0.00001 (for fine-tuning)

**Table 7:** Performance Comparison of Different Models for Kaggle Dataset.

Model	Accuracy	F1 Score	Precision	Recall
DenseNet121	0.95	0.95	0.95	0.95
InceptionV3	0.92	0.92	0.92	0.91
ResNet50	0.90	0.88	0.85	0.87
VGG16	0.87	0.86	0.80	0.85
Xception	0.85	0.85	0.87	0.86
MobileNetV2	0.83	0.84	0.83	0.84
NASNetMobile	0.81	0.80	0.82	0.80
EfficientNetB0	0.75	0.60	0.55	0.65
InceptionResNetV2	0.70	0.70	0.77	0.75
MobileNet	0.67	0.75	0.80	0.78
SmartSkin-XAI	0.98	0.96	0.97	0.97

**Table 8:** Performance Comparison of Different Models on the ISIC Dataset.

Model	Accuracy	F1 Score	Precision	Recall
DenseNet121	0.91	0.89	0.90	0.90
InceptionV3	0.88	0.86	0.87	0.88
ResNet50	0.85	0.83	0.84	0.85
VGG16	0.83	0.80	0.81	0.82
Xception	0.81	0.78	0.79	0.80
MobileNetV2	0.80	0.77	0.78	0.79
NASNetMobile	0.78	0.76	0.77	0.78
EfficientNetB0	0.74	0.71	0.72	0.73
InceptionResNetV2	0.71	0.68	0.69	0.70
MobileNet	0.68	0.65	0.66	0.67
SmartSkin-XAI	0.97	0.96	0.96	0.97

## Data Availability

The dataset used in this study, the Melanoma Skin Cancer Dataset, consists of 10,000 images and is publicly available on Kaggle. The dataset includes images labeled for the presence or absence of melanoma, enabling the training and evaluation of our skin cancer detection model. It can be accessed at the following link: https://www.kaggle.com/datasets/hasnainjaved/melanoma-skin-cancer-dataset-of-10000-images (accessed on 1 January 2022). The ISIC 2020 dataset used in this study is publicly available from the International Skin Imaging Collaboration (ISIC) Archive. It contains diverse, high-quality dermoscopic images for skin lesion analysis, widely recognized for research and clinical AI applications. The dataset can be accessed at the following link: https://doi.org/10.1038/s41597-021-00815-z (accessed on 28 January 2021).

## References

[1] Harahap M., Husein A.M., Kwok S.C., Wizley V., Leonardi J., Ong D.K., Ginting D., Silitonga B.A. (2024). Skin cancer classification using EfficientNet architecture. Bull. Electr. Eng. Inform..

[2] Renu I.Z., Haque M.M., Paul S.K., Mou M.S., Rahman M.N., Gupta S.S., Paul R.R. A Comprehensive Analysis on Skin Cancer Classification Using Transfer Learning. Proceedings of the 2024 3rd International Conference on Advancement in Electrical and Electronic Engineering (ICAEEE).

[3] Kachare K., Bhagat N., Raundale P. Advancements in Melanoma Skin Cancer Detection Using Deep Learning: A Comprehensive Review. Proceedings of the 2023 7th International Conference on Computing, Communication, Control and Automation (ICCUBEA).

[4] Thwin S.M., Park H.S. (2024). Skin Lesion Classification Using a Deep Ensemble Model. Appl. Sci..

[5] Sanjana C.V., Raju S.S., Anusha M., Sivani B. Classification and Detection of Skin Cancer Using Deep Learning Methods. Proceedings of the 2023 International Conference on Computer Communication and Informatics (ICCCI).

[6] Anil P., Narayana B.J.L., Reddy G.K.T., Choudhary S.R., Sri K.S. Skin Cancer Classification with DenseNet Deep Convolutional Neural Network. Proceedings of the 2023 4th IEEE Global Conference for Advancement in Technology (GCAT).

[7] Siddique A., Shaukat K., Jan T. (2024). An Intelligent Mechanism to Detect Multi-Factor Skin Cancer. Diagnostics.

[8] Salomi M., Daram G., Harshitha S.S. Early Skin Cancer Detection Using CNN-ABCD Rule Based Feature Extraction Classification and K-Means Clustering algorithm through Android Mobile Application. Proceedings of the 2024 Second International Conference on Emerging Trends in Information Technology and Engineering (ICETITE).

[9] Hamim S.A., Jony A.I. (2024). Enhancing Brain Tumor MRI Segmentation Accuracy and Efficiency with Optimized U-Net Architecture. Malays. J. Sci. Adv. Technol..

[10] Mittal R., Malik V., Singh J., Gupta S., Srivastava A.P., Sankhyan A. Skin Cancer Detection Using Deep Block Convolutional Neural Networks. Proceedings of the 2023 10th IEEE Uttar Pradesh Section International Conference on Electrical, Electronics and Computer Engineering (UPCON).

[11] Alshalman M., Gargoum B.F., Nagem T., Bozed K.A. Skin Cancer Detection by Using Deep Learning Approach. Proceedings of the 2023 IEEE 11th International Conference on Systems and Control (ICSC).

[12] Avanija J., Reddy C.C.M., Reddy C.S.C., Reddy D.H., Narasimhulu T., Hardhik N.V. Skin Cancer Detection using Ensemble Learning. Proceedings of the 2023 International Conference on Sustainable Computing and Smart Systems (ICSCSS).

[13] Prasad C.R., Bilveni G., Priyanka B., Susmitha C., Abhinay D., Kollem S. Skin Cancer Prediction using Modified EfficientNet-B3 with Deep Transfer Learning. Proceedings of the 2024 IEEE International Conference for Women in Innovation, Technology & Entrepreneurship (ICWITE).

[14] Hayat S.N., Indraswari R. (2024). Skin Cancer Detection Approach Using Convolutional Neural Network Artificial Intelligence. Int. J. Inform. Inf. Syst..

[15] Natha P., RajaRajeswari P. (2024). Advancing Skin Cancer Prediction Using Ensemble Models. Computers.

[16] Ahmed K.T., Rustam F., Mehmood A., Ashraf I., Choi G.S. (2024). Predicting skin cancer melanoma using stacked convolutional neural networks model. Multimed. Tools Appl..

[17] Malaiarasan S., Ravi R., Maheswari D., Rubavathi C.Y., Ramnath M., Hemamalini V. Towards Enhanced Deep CNN For Early And Precise Skin Cancer Diagnosis. Proceedings of the 2023 International Conference on Networking and Communications (ICNWC).

[18] Khamsa D., Pascal L., Zakaria B., Lokman M., Zakaria M.Y. Skin Cancer Diagnosis and Detection Using Deep Learning. Proceedings of the 2023 International Conference on Electrical Engineering and Advanced Technology (ICEEAT).

[19] Neeshma A., Nair C.S. Multiclass skin lesion classification using densenet. Proceedings of the 2022 Third International Conference on Intelligent Computing Instrumentation and Control Technologies (ICICICT).

[20] Yee J., Rosendahl C., Aoude L.G. (2024). The role of artificial intelligence and convolutional neural networks in the management of melanoma: A clinical, pathological, and radiological perspective. Melanoma Res..

[21] Lin T.L., Lu C.T., Karmakar R., Nampalley K., Mukundan A., Hsiao Y.P., Hsieh S.C., Wang H.C. (2024). Assessing the efficacy of the spectrum-aided vision enhancer (SAVE) to detect acral lentiginous melanoma, melanoma in situ, nodular melanoma, and superficial spreading melanoma. Diagnostics.

[22] Rey-Barroso L., Burgos-Fernández F.J., Delpueyo X., Ares M., Royo S., Malvehy J., Puig S., Vilaseca M. (2018). Visible and extended near-infrared multispectral imaging for skin cancer diagnosis. Sensors.

[23] Filippi L., Bianconi F., Schillaci O., Spanu A., Palumbo B. (2022). The role and potential of 18F-FDG PET/CT in malignant melanoma: Prognostication, monitoring response to targeted and immunotherapy, and radiomics. Diagnostics.

[24] Singla M., Gill K.S., Kumar M., Rawat R. Cutting-edge Dermatological Advances using Deep Learning for Precise Skin Cancer Classification. Proceedings of the 2024 International Conference on Smart Systems for applications in Electrical Sciences (ICSSES).

[25] Gouda W., Sama N.U., Al-Waakid G., Humayun M., Jhanjhi N.Z. (2022). Detection of skin cancer based on skin lesion images using deep learning. Healthcare.

[26] Gupta P., Mesram S. (2022). AlexNet and DenseNet-121-based hybrid CNN architecture for skin cancer prediction from dermoscopic images. Int. J. Res. Appl. Sci. Eng. Technol..

[27] Javed H. (2022). Melanoma Skin Cancer Dataset of 10,000+ Images. https://www.kaggle.com/datasets/hasnainjaved/melanoma-skin-cancer-dataset-of-10000-images.

[28] Rotemberg V., Kurtansky N., Betz-Stablein B., Caffery L., Chousakos E., Codella N., Combalia M., Dusza S., Guitera P., Gutman D. (2021). A patient-centric dataset of images and metadata for identifying melanomas using clinical context. Sci. Data.

